# The Story of the Insane from Year to Year

**Published:** 1905-10-14

**Authors:** 


### THE STORY OF THE INSANE FROM YEAR TO YEAR.

Seventeenth Annual Series.

### WEST SUSSEX ASYLUM, CHICHESTER.

On the first of January this asylum contained 728 patients, and on the last day of the year the number was reduced to 659, such reduction being due to the removal of out-county patients to their own asylums. There were 123 first admissions; 28 readmissions; 47 recoveries; 37 discharged relieved; 77 discharged not improved, and 59 deaths. The total number under treatment during the year was 879, and the average daily number resident was 695. The recoveries stand at 32·19 per cent. of the admissions and the deaths at 8·92 per cent. of the average number resident. Of the year's deaths 32 were caused by cerebral and spinal diseases and 9 by consumption. Various moral causes account for the insanity in 19 of the patients admitted; intemperance in drink was the cause in 24; hereditary influence in 63, and previous attacks in 38. There were 19 in whom no cause was known. Of the 151 cases admitted during the year Dr. Kidd can only find 30 in whom recovery is probable, and 39 in whom it is possible, which fairly well shows that the raw material he has to work on is not very promising for a high percentage in his next year's recovery table. It is most unfortunate that applications for admission of private patients had to be declined, as there was a lack of special accommodation for them; and we much regret to learn that the Committee have fixed £1 1s. a week as the lowest rate of board for private patients, as such rate should be the very highest asked for in a county asylum. It is to be hoped that the Committee will reconsider their decision on this matter, and enable many patients to avoid that "stigma of pauperism" referred to in the Report, as well as reduce the sums payable by the parish authorities. We further regret that the Committee have not seen fit to formulate a Pensions Scheme for their staff. Surely the subject is important enough. This asylum is only about seven years old, and yet we are told in this Report that £2,700 have to be spent on an increased water supply, £616 on storage batteries, and that the heating apparatus of the chapel is insufficient. The asylum possesses no medical reference library and no operating room. The weekly rate of maintenance is 12s. 10d.

